# Change management in hospital digital transformation: A roadmap to support planning and implementation for humanisation and efficiency through technology

**DOI:** 10.1371/journal.pone.0344555

**Published:** 2026-04-09

**Authors:** Juliana Basulo-Ribeiro, Francisco Rocha-Gonçalves, Alberto Freitas, Leonor Teixeira

**Affiliations:** 1 Department of Economics, Management, Industrial Engineering and Tourism (DEGEIT), Institute of Electronics and Informatics Engineering of Aveiro (IEETA), Intelligent Systems Associate Laboratory (LASI), University of Aveiro, Aveiro, Portugal; 2 Department of Community Medicine, Information and Health Decision Sciences (MEDCIDS), Faculty of Medicine, University of Porto, Porto, Portugal; 3 CINTESIS – Center for Health Technology and Services Research, Faculty of Medicine, University of Porto, Porto, Portugal; Pontificia Universidad Catolica de Chile, CHILE

## Abstract

Digitalisation offers promising solutions for improving efficiency and accessibility in healthcare. However, it is crucial to balance digital advancements with humanisation. The aim of this study is to support digital transformation in healthcare by striking a balance between the digital and human components, by proposing a roadmap for digital transformation, prioritizing people and organisational culture. An initial theoretical and contextual investigation guided the preliminary roadmap, addressing concerns and solutions from the literature. Then, an empirical study was conducted via interviews with Portuguese hospital managers, offering practical insights into initiatives for hospital digitalisation. These findings refined the roadmap with real-world perspectives. The roadmap is presented as a theoretically informed and empirically refined planning proposal; it has not yet been piloted in real-world settings or compared with existing implementation frameworks. Managers highlighted the human dimension as critical to successful digital transformation, reinforcing the need for such a roadmap, an integrated approach that considers technology, processes and people within organisational culture. Humanisation is treated as a measurable target, monitored through patient-experience and staff-experience indicators. Theoretically, this study advances the literature on change management and digital transformation in healthcare. Practically, it offers an exploratory roadmap to guide healthcare organisations in structuring, sequencing and monitoring digital transformation initiatives. Transferability to other contexts requires local tailoring and future piloting with broader stakeholder involvement.

## 1. Introduction

### 1.1 Background

The concept of a VUCA world, characterized by volatility, uncertainty, complexity and ambiguity that emerged after the Cold War, has given way to the notion of a BANI world (introduced by the Covid-19 pandemic), which highlights the brittleness, anxiety, non-linearity and incomprehensibility of our times. This change underlines how reality is currently unpredictable and that the only certainty we have is change (“change is the new normal” [1]) [2].

The accelerated urbanization of the global population poses considerable challenges to conventional health systems, making the task of meeting citizens’ needs progressively more challenging [3]. Thus, Health 4.0 is emerging as a crucial element in future socio-economic planning, promoting multiple benefits for the health system [4].

In the European context, and particularly in Portugal, digitalisation is being promoted as a solution to make the health sector more efficient and accessible, potentially generating significant economic benefits [5]. However, Huaytan et al. [6] point out that any digital transformation strategy must be comprehensive and coordinated, encompassing technological, regulatory, educational and financial aspects. The accomplishment of smart health depends on various components, such as smart ambulance systems, smart hospitals, wearable devices and emergency response [7], enabling connected medical devices and smart systems to promote diagnostics, health monitoring and well-being [3].

Thus, digitalisation has emerged as a promising “remedy” for increasing efficiency and accessibility in the health sector. However, it is necessary to digitise healthcare without losing humanity. According to Chaabi [8], the focus of Industry 4.0 is the automation of processes and the integration of new technologies, but in contrast, Industry 5.0 shifts the emphasis to a human-centred model, repositioning human workers at the centre of the production process [9].

As Feki and Boughzala [10] state, “digital transformation is more than just technological change”. Digital transformation in healthcare is therefore a phenomenon that goes beyond mere technological change; it is a reconfiguration of the practices and structures that form the heart of healthcare. We are witnessing an evolution that changes the way healthcare is designed, delivered and managed. The Covid-19 pandemic has accelerated this process, highlighting the urgent need for innovation and the adoption of new ways of providing health services, facilitating diagnoses and ensuring data analysis, as the studies by Dal Mas et al. [11] and Megawati et al. [12] illustrate.

Furthermore, Wanasinghe et al. [13] discuss the concept of human-centred digital transformation, emphasizing the importance of prioritizing the humanisation in digital initiatives. These insights highlight the need to incorporate human-centred principles on the path to digital transformation in healthcare to ensure that the needs, competencies and perceptions of healthcare professionals and stakeholders are adequately addressed throughout the process [14]. In line with this topic, research by Jarva et al. [15] highlights the importance of healthcare professionals’ perceptions of digital health competence, emphasizing the importance of understanding and addressing the skills and competences required for a successful digital transformation.

The centrality of the human being in digital transformation is not only an ethical issue, but also a strategic one. Cutting-edge technologies, no matter how advanced, need a human touch to ensure that they are effectively adopted and that they really add value to processes in the specific context of healthcare. This means creating systems that are intuitive, accessible and that improve interaction between healthcare professionals and patients. Thus, this balance between technology and humanity is fundamental to building trust and for digitalisation to be truly transformative. Adopting practices that promote digital inclusion and ensuring that all healthcare professionals have the necessary skills to use the new technologies, are crucial steps in this process [16]. In this way, it can be ensured that hospital digital transformation is carried out in an inclusive and equitable manner, paving the way for more personalized and patient-centred healthcare [17–19].

We are moving towards a Society 5.0, which aims to address social issues to raise people’s standard of living. Achieving this goal will be facilitated by technology and the transition to a socio-economic system that prioritizes the recognition and appreciation of human and environmental capital, to the detriment of the constant search for profits [20]. As mentioned by Ciasullo et al. [4] the concept of Health 4.0 establishes a model for the provision of health services that is consistent with the principles underlying Society 5.0. In this context, Health 4.0 aims to involve professionals and users in making the most of the intellectual capital available to healthcare organisations, with the aim of structuring health promotion and risk prevention services that are consistent with a patient-centred approach to care [21]. To achieve this goal, hospitals must adapt to this paradigm shift, and it is crucial to understand how the challenges associated with adopting new technologies in the hospital environment can be mitigated.

Angerer et al. [22] state in their study that for this paradigm shift to digital in the hospital context to be successful, close collaboration between managers and technology specialists is essential, focusing not only on technology, but also on its implications for the strategic and operational management of healthcare entities. As a result, it was concluded that hospital digital transformation requires a holistic approach that transcends the simple implementation of new technologies. A well-defined approach that integrates technological innovation with healthcare processes, organisational culture and the patient experience itself is essential.

### 1.2 Study motivation

As we have seen so far, it is common sense that digital transformation brings benefits in general to all areas, especially in the health sector, not only for the patients themselves, but also for health organisations (both on the “demand” and “supply” side).

The results of a previous study based on interviews with 11 health professionals [23] reinforced the importance of this research, contributing to the motivation behind this study. In that study, health professionals stated:

- that resistance to change on the part of health professionals and low digital literacy are the most common challenges that usually prevent technological adoption in the health sector; this is also argued by several other studies [16,18,24];
- for the digital transformation to be successful, it is imperative to have multidisciplinary collaboration between the various health professionals and IT professionals, to optimize the use of these technologies in the provision of healthcare, thus emphasizing the importance of people, as it is recognized by [25]; and
- correctly identifying the problems to be solved implies working on processes to develop robust and effective technologies from the point of view of improving health outcomes and the efficiency and effectiveness of services.

Therefore, for the digital transformation to take place in the best way, a balance is needed between three essential pillars: people, processes and technology, guided by cultural aspects, which also reinforces the importance of studying culture. As highlighted by Gomathi & Mishra [18] in their study, the interaction between humans and technology aims to improve the efficiency and effectiveness of production processes. Furthermore, according to Paul & Zhou [26], “Having the right balance of people, process, and technology has always been the key to the success of any transformation and the core of company competitiveness, as well as the innovation capability”.

People and processes are often (mistakenly) “neglected” during this shift to the digital, despite being crucial elements of this transformation, as highlighted by the healthcare professionals interviewed. So it is in this area that this article aims to contribute, with a tool that makes it possible to manage the digitalisation process while keeping people at the heart of development. In line with this thinking, Gilli et al. [27] state in their study that, more than technology, people and their culture drive digital transformation. Furthermore, according to Konttila et al. [28], health sector organisations must focus on social dynamics in the workplace and foster a positive environment to improve their adaptability to digital transformation.

**Fig 1:**
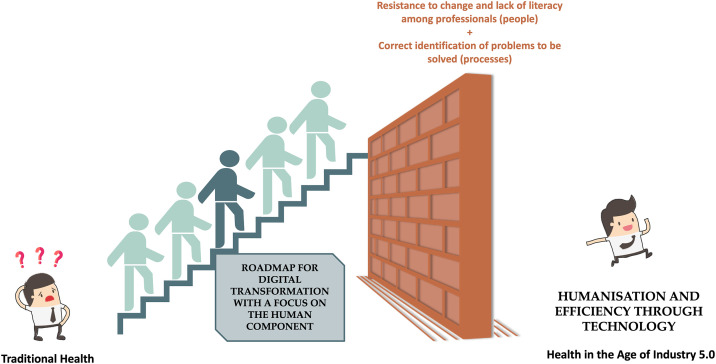
Study motivation.

Thus, there is a need for a guide to support hospitals in planning and implementing digital transformation initiatives, focused on people and leveraged by processes, can facilitate the adoption of and adaptation to new technologies in the hospital environment, and this challenge is the subject of this study (see Fig 1).

## 2. Theoretical background

### 2.1 Organisational culture management and organisational behaviour

Organisational culture, defined as a system or set of values, beliefs and behaviours (feeling, thinking, acting, etc.) that shape the way members of an organisation interact with each other and with the outside world, is a crucial factor in operational success [29–31]. “Culture is to the organisation what personality is to the individual” [32].

On the other hand, everyone brings with them a set of beliefs, which are shared between different individuals through social interaction Castellano as cited in [33]. These beliefs may or may not be shared by everyone, however; according to Grabovetter as cited in [33] individuals are predisposed to change their minds if a larger group around them holds that other belief.

Furxhi [34] points out that employees’ reactions to change are fundamental, since they are the ones who implement it. On the one hand, a (negative) culture that shows readiness for change or resistance to change and remains attached to outdated procedures can hinder the process of change in the organisation. Some authors argue that resistance to change can be as much an organisational and group issue as an individual one and is caused by factors such as: fear of the unknown, lack of communication and involvement, and bureaucratic organisational structures [34,35]. On the other hand, a positive organisational culture, which values continuous improvement, innovation and collaboration, can facilitate the implementation of practices that lead to desirable operational outcomes, such as the efficiency and effectiveness of operations [30].

Organisational culture influences employee motivation and satisfaction, organisational performance and cognitions, and has a direct impact on organisational behaviour [33,36]. Organisational behaviour can be defined as: the field of study in which the aim is to investigate how the individual behaviour of each individual impact’s behaviour within the organisation, in order to increase operational efficiency [37,38]. The dedication and involvement of people within an organisation is crucial for operational excellence, so it is important to create a work environment that motivates employees [39]. Thus, it is important to develop a culture that values not only social aspects, but also cognitive ones, creating an environment that encourages positive and productive behaviours, such as: helping colleagues, being punctual, and following company rules in a way that promotes a positive and efficient work environment [33,36,38].

The effective management of organisational culture is a complex and multidimensional challenge faced by leaders and managers. With the growing understanding that an organisation’s culture can be a catalyst or inhibitor to its success, the need to adopt robust cultural management models has become imperative.

Van Der Post et al. [32] identified 15 culture constructs in their study which allow it to be measured: conflict resolution; culture management; customer orientation; disposition towards change; employee participation; goal clarity; human resources orientation; identification with organisation; locus of authority; management style; organisation focus; organisation integration; performance orientation; reward orientation; task structure.

There are several models in the literature that measure organisational culture. However, the literature points to several difficulties in measuring it, as it is complex and multifaceted. These include: the concept of organisational culture being made up of a huge set of interrelated norms; the lengthy process due to having several levels; that selection bias can occur, triggered by the attempt to convey a positive image of the company (even if it is not entirely true); and the lack of construct validity of existing scales [33,40,41]. Table 1 shows some of the organisational culture management models in the literature.

**Table 1:** Organisational culture management models.

MODEL	EXPLANATION	CITATIONS
Edgar Schein’s Organisational Culture Model	Model using three levels of cultural analysis: 1. Artifacts: The tangible elements of a company’s culture. They include things from the physical layout of a workspace to the dress code, and can extend to company rituals, the way employees interact and even company slogans. Although these are the most visible aspects, they can be difficult to interpret because their cultural significance is not immediately apparent. 2. Expressed values: This layer concerns the expressed values and norms that are communicated verbally within an organisation. These represent the ideal culture that the organisation strives to achieve and are the justification for certain behaviours or practices. 3. Basic assumptions: This deeper level consists of unconscious beliefs and values that truly define the essence of the organisational culture. They dictate behaviour, perception and thinking and are therefore the most difficult to change. To understand how a company behaves, you need to understand these assumptions.	[42–44]
Denison’s Organisational Culture Model	Model focused on four cultural traits, interconnected with the organisation’s performance: 1. Mission: Clarifying the organisation’s purpose and direction through vision, goals and objectives. 2. Consistency: Capturing the organisation to coordinate activities, reach agreement and integrate efforts. Includes core values, agreement and coordination. 3. Involvement: Capturing the organisation to encourage team orientation and develop employees’ skills. 4. Adaptability: The organisation’s ability to translate external changes into action, highlighting the importance of customer focus, organisational learning and creating change.	[44–46]
O’Reilly, Chatman & Caldwell’s Organisational Culture Profile (OCP)	Instrument capable of measuring seven dimensions of organisational culture, related to the values and norms of the organisation: 1. Innovation: Reflects a culture that emphasizes novelty and creativity. *2.* *Outcome orientation:* Focuses on results and achievements, highlighting a culture that values performance and meeting objectives. *3.* *Aggressiveness:* Characterizes a culture that is competitive and motivated, often focused on outperforming competitors. *4.* *Detail orientation:* Denotes a culture that values precision, analysis and attention to detail. *5.* *Team orientation:* Indicates a culture that values collaboration, teamwork and a sense of unity among employees. *6.* *People oriented:* Describes a culture that values fairness, support and respect for the individual. *7.* *Stable:* Relates to a culture that emphasizes consistency, predictability and maintaining the status quo.	[44,47,48]
The Competing Values Framework by Cameron and Quinn	A model that divides organisational culture into four main types, spread over two axes (stability and flexibility): 1. Clan culture: In this type of culture, group cohesion and morale are priorities, facilitating the development of human resources. 2. Culture of adhocracy: This culture emphasizes innovation as a way of growing and adapting to the constant changes in the market. 3. Market culture: Focuses on competition and achieving results, with an emphasis on clear and targeted goals and objectives. 4. Hierarchical culture: Uses a clear and well-defined structure and procedures, with formal communication and centralized decisions to ensure efficiency, order and organisational stability.	[49–51]

In addition to these four models, there are many others, such as: culture audit (an assessment method that can include workshops, interviews and questionnaires, among others, to understand organisational culture); Hofstede’s cultural dimensions theory (a model that helps to understand cultural differences within the company); and the organisational culture assessment instrument (OCAI); among many others [52,53]. These models make it possible not only to identify and understand the underlying values and rules that guide and influence people’s behaviour in an organisation, but also to offer a strategy for involving people and applying changes that bring the culture into line with corporate goals.

The assessment and management of organisational culture in hospitals is an essential part of the healthcare system [54]. This is a crucial process for understanding how current beliefs and behaviours reinforce or hinder the achievement of patient care goals and operational efficiency. This cultural assessment, more than a measurement of employee satisfaction, is a comprehensive diagnosis that seeks to align daily practices with the goals of the health service. By identifying behavioural patterns and underlying values, hospital leaders can implement changes that not only improve the patient experience, but also optimize processes, leading to improvements in performance and organisational performance [29,55]

### 2.2. Outcomes in health and organisational culture

The quality of healthcare is a constant concern around the world, due to the need to ensure sustainability, control costs, and promote effective, safe and patient-centred care. These measures aim to increase transparency and accountability and improve health outcomes and patient satisfaction [56,57].

To effectively evaluate organisational change, it is crucial to measure health outcomes, which can be classified into different groups. The WHO [58] identified six key dimensions for assessing hospital performance: clinical outcomes; production outcomes; patient centredness; responsive governance; staff orientation; and safety. Table 2 below shows these same groups and the type of metrics that can be measured in each of them. This data allows adjustments to be made on an ongoing basis to enhance the quality of health services.

**Table 2:** The six key dimensions of hospital performance.

Outcome Types	Explanation	Metrics
Clinical outcomes	This refers to the efficient use of resources to optimize health outcomes, guaranteeing the correct application and safety of the care processes themselves.	Technical quality, evidence-based practice and organisation, health improvement and results (both individual and patient-related).
Production outcomes	Effective and optimized allocation of available health resources to maximize clinical and operational results, which improves productivity and capacity utilization within the hospital structure. Specifically, it covers the adequacy of the services provided, the effectiveness of clinical processes, and an organisational structure that supports these same processes.	Resources and financial components (financial systems, continuity, additional resources), more highly qualified staff and the use of state-of-the-art medical equipment, technology and techniques.
Patient centredness or focus on patient	This assesses the orientation towards patients and their families, gauging whether patients are at the centre of the delivery of care and services.	Availability to patients: focusing on the client (immediate attention, access to social assistance, quality service provided, selection of service provider), patient satisfaction and patient experience (dignity, confidentiality, autonomy, communication).
Social accountability or responsive governance	This assesses the degree of response to the needs of the community, to ensure continuity and coordination of care, promote health and provide care for all citizens.	Community orientation (response to needs and demands), access to resources, continuity, health promotion, equity, ability to adapt to the growing demands of the population (strategically).
Staff orientation	Meeting the growing demand for human resources, implementing incentive strategies to retain qualified professionals, such as doctors and nurses, and providing a safe and supportive working environment for their professional development. All of this includes: ensuring equitable access to continuing medical education, recognizing individual needs, promoting health and implementing effective safety measures.	Health, well-being, satisfaction, development (e.g., turnover, vacancies, absence).
Safety	Assess whether patients are satisfied with medical services and whether providers are aware of the need to establish effective collaboration with the hospital, within an efficient organisational structure. This applies to both patients and healthcare professionals, emphasizing the ability to avoid, prevent and minimize interventions which may be harmful to them or environmental risks.	Safety of patients and providers, structure and process.

Adapted from: [58] | Information taken from: [56–60]

As seen above, organisational culture is crucial within an organisation and plays a critical role in determining health outcomes (key performance indicators) [61]. A strong culture that values collaboration, innovation and commitment can positively influence operational efficiency, patient satisfaction and staff motivation. Furthermore, it can be stated that in environments where transparent communication, mutual support, and continuous learning are encouraged, there is a greater likelihood that processes will be optimized and errors minimized, thus improving clinical outcomes. [29,61–64]. Mannion & Davies [29] even point out that organisational culture is often cited as the culprit when health scandals arise, and cultural change is also prescribed as a remedy to solve the problems.

Therefore, investing in organisational culture not only boosts overall performance, but also promotes an environment where professionals feel valued and patients consequently receive higher quality care. For this very reason, organisational culture is a crucial vector for driving significant improvements in overall health and well-being [29,63–66].

Thus, it can be concluded that understanding and shaping organisational culture is essential for healthcare leaders seeking to improve operational results and promote a more effective and safer work environment. Braithwaite et al. [65] suggest that healthcare administrators and policymakers should focus on cultivating and promoting positive organisational cultures to improve hospital performance.

### 2.3 Change management

Change management is the method adopted by an organisation with the aim of recognizing new demands and/or limitations imposed by the external environment and adapting to them. It also involves determining the strategic and operational initiatives considered crucial to optimizing organisational performance. It encompasses planning, executing and evaluating appropriate initiatives to ensure the continued success of the organisation [67].

Lichem-Herzog & Sorko [68] argue that without a well-defined plan, companies have difficulty in change processes. Gilli et al. [27] state in their study that the digital transformation is no different from other changes at the organisational level, arguing that existing knowledge about change management is applicable to the introduction of the digital paradigm.

Hubbart points out that an organisation’s culture significantly influences its agility and ability to adapt to changing operations, which are crucial to operational success, further stressing that resistance to change is not only a barrier, but also an instinctive reaction that can be mitigated through proactive leadership and inclusive practices [35].

It is therefore essential to understand the causes of employee resistance so that managers can design effective change processes that achieve the desired results. In this context, according to Prosci, a global organisation specialized in change management, organisational culture is one of the main causes of resistance to change, including aspects such as risk-averse cultures, past negative experiences with change, and issues of mistrust between departments and hierarchical levels [69]. Furthermore, as stated by Hubbart [35], the sources of resistance to change among workers include scepticism and insufficient confidence, emotional reactions, apprehension of failure, ineffective communication and time constraints. More are identified: expectations generated from previous experiences, leaving the comfort zone which can generate discomfort, and fear of change [1,70].

The literature presents a series of strategies aimed at developing the culture of organisations, reducing resistance to change and, consequently, improving operational results [34,35]. These techniques involve both the understanding and harmonization of corporate values and practical measures for the continuous improvement of operational processes. Thus, effective strategies for overcoming this resistance and managing change emphasize the importance of transparent communication, the participation of all people in decisions related to change, the alignment of organisational culture and structure with the desired change, and an active leadership role to mitigate employee resistance to change [69]. Below are different strategies for successful change:

- Effective communication: It is essential to clearly communicate the purpose, benefits and expected impacts of the changes to be implemented, as this can help align employees with organisational objectives and reduce resistance to change (an inhibiting factor). Furthermore, according to Lichem-Herzog & Sorko [68] “70% of all change processes fail due to the lack of a clear goal and a systematic approach to change” [35,67].
- Participation and involvement: It is crucial to involve employees in the change process, from planning to implementation, so that acceptance and commitment to the proposed changes can be increased. Involving employees in change initiatives can lead to a greater sense of ownership and stronger alignment with organisational goals. Collaboration and cooperation between people is essential for change to be successful, and the transition must take place in an environment of dialogue, trust and reciprocal agreement, rather than being unilaterally imposed on the members of the organisation [35,71–74].
- Training and development: Training and development serve to empower employees with the skills necessary for change, enabling people to be effective in the workplace. Providing adequate training can help people feel less threatened and more prepared for change, facilitating this transition [72,74].
- Supportive leadership: Leaders who demonstrate support during the change process and who are committed to continuous improvement can positively influence organisational culture, encouraging acceptance and adaptation to new practices [27,35,73,74].

When organisations are successful in their change processes, they see effort and dedication from their people to the new guidelines, experience fewer fluctuations in productivity throughout the implementation phase, and achieve the completion of changes in considerably shorter periods of time [67]. We can therefore conclude that there is a relationship between effective change management and health outcomes.

#### 2.3.1 Main change management models.

Change management is therefore a methodology that combines strategies and tactics to promote and facilitate effective change processes [75]. There are various methods used to facilitate change in an effective and sustainable way. Errida & Lotfi [76] analysed 37 organisational change models in their study. Table 3 below gives three example models.

**Table 3:** Change management models.

MODEL	EXPLANATION	CITATION
ADKAR	The ADKAR approach serves as an effective approach for managing changes within organisations. The five phases are: • Awareness: Understanding the need for change and the benefits of this change; • Desire: Fostering a personal commitment to support and getting involved in change, creating the desire to change; • Knowledge: Providing the information and training needed for change, understanding the needs of professionals; • Ability: Developing the skills and behaviours needed for change to take place; • Reinforcement: Ensuring that change is sustained through positive reinforcement and support, i.e., providing support during the new stage (ensuring that the new practices are sustained and are part of the organisation’s culture).	[26,75,77,78]
Kotter	Kotter proposes an eight-step model to guarantee a successful change process: 1. Establish a sense of urgency: Communicate the immediate need for change. 2. Form a guiding alliance: Bring together a group with the power and influence to lead the change. 3. Develop the vision and strategy: Create a clear vision for the change and strategies to achieve it. 4. Communicate the vision of change: Ensure that the vision is transmitted and understood by everyone in the organisation. 5. Empower large-scale action: Remove obstacles and enable people to act in line with the vision. 6. Celebrate short-term victories: Plan and achieve quick results that reinforce the change process. 7. Consolidate gains and produce more change: Use the credibility gained to change systems, structures and policies that are not aligned with the vision of change. 8. Sustain the new approaches in the organisational culture: Ensure that the new practices are maintained and become part of the organisational culture.	[75,76,79]
Lewin	Lewin’s model is considered the theoretical basis for change management. This model involves three main stages: 1. *“Unfreezing”*: This involves destabilizing the current state to foster recognition and support for the need for change, preparing the ground for the new way of operating. 2. *“Transition”*: This refers to implementing the planned changes, moving towards the desired state. 3. *“Refreezing”*: This takes place after the changes have been implemented, solidifying the new organisational culture with the new behaviours and practices.	[76,80,81]

Other change management models include: the change formula of Beckhard and Harris; McKinsey’s 7-S framework; Bridges’ model of transition; Garvin and Roberto’s change model; General Electric’s change model; and many more, many of which are combinations of each other [76,82].

#### 2.3.2 The ADKAR model.

Among the several change management frameworks identified in the literature, the ADKAR model was selected for this study because it offers a practical and people-centred structure that directly aligns with the human dimension of digital transformation. While other organisational approaches such as Kotter’s 8-Step model or Lewin’s three-stage process place greater emphasis on organisational structures, leadership or strategic alignment, ADKAR is specifically designed to support change at the individual level [83,84].

By emphasising individual behavioural progression through the sequential stages of Awareness, Desire, Knowledge, Ability and Reinforcement, ADKAR provides a clear and operational tool to understand how people adopt and sustain new practices. This characteristic makes the model particularly suitable for contexts in which the success of digital initiatives depends heavily on human factors. Given that the successful adoption of digital technologies in hospitals depends strongly on staff engagement, readiness and capability building, ADKAR provides a clear and actionable lens for analysing the human side of transformation.

In healthcare, change management is even more crucial due to the specialized nature of clinical work and the complexities inherent in the sector. This area is fundamentally about people, from healthcare professionals to users, which further exacerbates issues of resistance to change and sometimes, given the diversity of people involved, a lack of digital literacy. Digital transformation requires adapting traditional methods, which can be a challenge as it not only alters the provision of care, but also requires new skills. Therefore, the adoption of change management methodologies is essential to facilitate a smooth transition to these new practices and to fully reap the benefits of digitalisation.

In his study, Chaabi [8] used the ADKAR methodology, combined with another, to establish a roadmap for implementing I5.0 in the context of industry. Also, in a study by Babin & Ghorashy [85], the authors conclude that incorporating the ADKAR model into the change management process significantly improved the success of their innovation initiatives. Specifically, they found that integrating structured change management using this model during the implementation of digitalisation initiatives led to better acceptance and understanding of the change by the entire organisation, highlighting the importance of addressing the human side of change to ensure the successful adoption and sustained benefit of new technologies. The author of the model himself writes in his book: “the model provides a framework and sequence for managing the people side of change”.

ADKAR, a model developed by Hiatt [78] is an acronym representing a structured approach for organisational change management, guiding individuals, and teams through five sequential phases: awareness, desire, knowledge, ability, and reinforcement, facilitating a gradual and successful transition to desired changes within a company or project [77]. According to Paul & Zhou [26], the ADKAR approach serves as an effective strategy for managing changes within organisations, offering a roadmap to assess and rectify resistance, with a focus on planning and executing tailored actions that underpin effective change and ensure the achievement of goals. According to Boca [77] the aims of the five phases are:

- **Awareness** – Understanding the need for change and the benefits of this change;
- **Desire** – Fostering a personal commitment to support and get involved in change, creating the desire to change;
- **Knowledge** – Providing the information and training needed for change, understanding the needs of professionals;
- **Ability** – Developing the skills and behaviours needed for change to take place;
- **Reinforcement** – Ensuring that change is sustained through positive reinforcement and support, i.e., providing support during the new stage.

**Fig 2:**
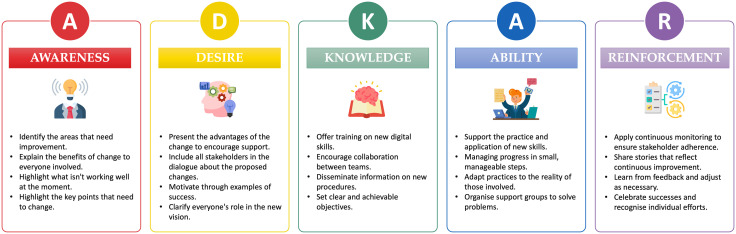
ADKAR methodology.

Fig 2 shows each of the phases of the ADKAR methodology.

Digitalisation is accepted by healthcare professionals when they understand that it helps to improve the quality of care and supports workflow processes. However, negative experiences, together with a lack of competence, cause frustration and a lack of desire to integrate the digital transformation process [28]. Effectively managing the complexity inherent in digital transformation requires focusing on the mindset and skills of employees. The necessary changes in processes, technologies and strategies can be achieved through training and professional development initiatives, internal communication, and systems that recognize and reward achievements [86].

Organisational culture, change management and healthcare outcomes are interconnected elements that jointly shape the success of digital transformation in hospitals. A supportive organisational culture creates the conditions for openness, collaboration and readiness for change. Change management provides the structured processes, such as communication, training and engagement, needed to translate this cultural foundation into the effective adoption of new technologies. When these two dimensions work together, digital initiatives are more likely to enhance both operational efficiency and human-centred care delivery. Thus, the interaction between culture and change management acts as a catalyst for improved healthcare outcomes in digital health initiatives. [87–91]

Building on this interaction, the balance between humanisation and digitalisation is also central to the theoretical foundation of this study. In this context/study, humanisation refers not only to empathy, communication and dignity in patient care, but also to the support, wellbeing and meaningful engagement of healthcare professionals who must adopt and work with new technologies. Digitalisation, in turn, focuses on efficiency, automation and data-driven processes. Rather than being opposing forces, these dimensions complement each other when digital tools are implemented through approaches that prioritise staff engagement, adequate training, and patient-centred and staff-centred workflows. This perspective underpins the contribution proposed in this study, illustrating how technology can enhance both patient care and the professional experience when supported by effective change management.

To make this concept operational within the roadmap and its measurement layer, “humanisation” is defined through observable dimensions at two levels: (i) patient experience (dignity, communication, autonomy, trust, and relational continuity) and (ii) staff experience (wellbeing, perceived workload, meaningfulness of work, psychological safety, and participation in decision-making). In this study, digitalisation is considered aligned with humanisation when it reduces administrative friction and supports more time and higher-quality clinical interaction, supports coordination, and strengthens autonomy and digital inclusion/capability without increasing inequalities or excluding groups.

## 3. Methods

### 3.1. Aims and goals

The aim of this article is to propose a roadmap to support the planning and implementation of hospital digital transformation, with a focus on the human dimension and organisational culture. Positioned as a change-management tool, it helps hospitals managing the transition towards digital transition through cultural adaptation and stakeholder engagement. The roadmap sets out a sequenced set of actions and associated KPIs to guide implementation and monitor progress, while foregrounding humanisation, staff engagement and cultural alignment. Its use requires local tailoring to organisational context and digital maturity.

To address this aim, the following research question was defined:

(RQ): *Which elements should a human-centred roadmap include to support the planning and implementation of hospital digital transformation, considering organisational culture and change management?*

Subsequently, and to ensure a more structured research process, the main research question was divided into two sub-questions:

i RQ1 (theoretical and contextual): *Which roadmap elements are supported by the literature and enriched by contextual expert inputs (observations and informal discussions)?*
ii RQ2 (empirical): *How do Portuguese hospital managers’ insights refine or reprioritise these elements?*

### 3.2 Methodology

**Fig 3:**
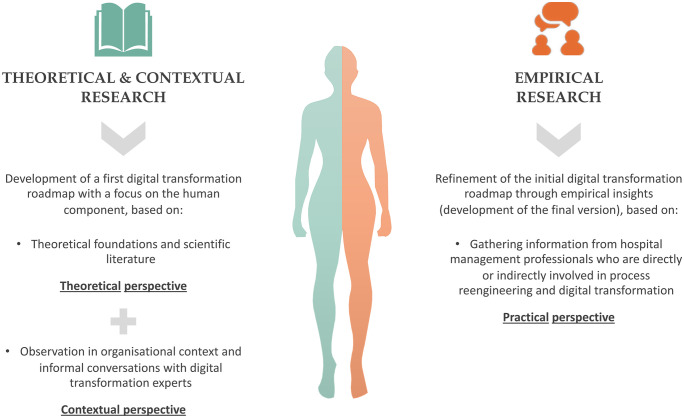
Methodology.

To achieve the study objective, a mixed-method research design was adopted, integrating both theoretical and empirical approaches (see methodology in Fig 3).

In the theoretical approach, the essential components for defining a digital transformation roadmap with a focus on the human dimension were identified. This stage combined (i) a structured literature review of change management, digital transformation, and organisational culture frameworks and (ii) practical observation in real organisational settings and informal conversations with domain experts involved in digital transformation projects.

This combined analysis allowed the initial version of the roadmap to be structured, outlining the fundamental pillars for successful and human-centred digital transformation in the healthcare sector.

The empirical approach aimed to refine the preliminary framework with insights from practice. To do this, eight semi-structured interviews were conducted with hospital management professionals involved in digital transformation in Portuguese hospitals. These interviews offered a practical view of ongoing initiatives and allowed the analysis of different digital transformation approaches. The findings helped identify best practices, barriers, and lessons learned, which supported the refinement of the theoretical model and the development of the final roadmap.

Thus, the final version of the roadmap emerged from the triangulation of evidence drawn from multiple sources: theoretical foundations, expert knowledge, and empirical evidence. This process helps to ensure that the model is conceptually grounded and contextually informed, reflecting implementation considerations reported in Portuguese hospitals while maintaining theoretical robustness.

For clarity, the methodology is presented in two parts: first, the development of the initial roadmap (based on the structured literature review and contextual inputs), and second, the development of the final roadmap (refined through semi-structured interviews with hospital managers).

#### 3.2.1 First roadmap development: Literature review and contextual inputs.

The data used to develop the initial version of the roadmap were first gathered through a structured literature review and observation in organisational context and informal conversations with digital transformation experts.

A structured literature review was conducted to identify key concepts and frameworks related to (i) hospital/healthcare digital transformation, (ii) change management, and (iii) organisational culture and human-centred transformation. Searches were performed in Scopus, PubMed and Web of Science between January 2024 and July 2024, using boolean combinations of keywords related to digital transformation/digital health, hospital/healthcare, and change management/culture/human-centred approaches. In addition, snowballing was used (screening reference lists and citations of key papers) to capture highly relevant studies not retrieved through database searches. Inclusion criteria comprised publications in English with explicit relevance to organisational implementation/change in healthcare digital transformation, primarily published from 2015 onwards. Seminal and methodological references published before 2015 (e.g., foundational change management and organisational culture frameworks or measurement instruments) were also included when directly relevant to the study constructs. Exclusion criteria comprised papers without relevance to organisational change. Screening occurred in two stages (title/abstract and full text).

To complement the literature review and strengthen contextual relevance, exploratory inputs were gathered through practical observation and informal engagement with digital transformation experts. Observations were carried out across four organisational settings between 2020 and 2023, allowing the researchers to capture how digital initiatives unfolded in real-world contexts. In addition, the research team held informal conversations with a broader pool of experts during this period; from these, seven longer and more detailed conversations were held with key informants (e.g., digital transformation leads, health IT managers, and clinicians involved in implementation), typically lasting 2–3 hours. These exploratory inputs were used to refine the preliminary roadmap’s structure, clarify terminology, and assess practical feasibility.

The initial roadmap was developed by analysing insights from the literature and the observations and informal discussions with digital transformation experts.

#### 3.2.2 Final roadmap refinement: Semi-structured interviews with hospital managers.

***3.2.2.1 Data collection procedures*:** Only after the theoretical and contextual foundation was established were semi-structured interviews conducted to complement and refine the preliminary framework.

To refine the roadmap with a practical perspective, a qualitative approach was chosen, which included semi-structured interviews with a group of experts, various hospital management professionals from various regions from the north to the south of Portugal. These interviews were carried out between May and June 2024, following pre-designed questions according to the established objective and the different phases of the ADKAR methodology, resulting in an interview script with ten questions. In addition to these questions, those introducing the interviewee and those dealing with consent to confidentiality and anonymity were also asked. Table 4 shows the objectives of each group of questions, distributed according to the ADKAR methodology.

**Table 4:** Objectives of each group of questions according to ADKAR methodology.

ADKAR phases	Explanation
**Awareness**	To identify the current state of awareness of digital transformation among hospital managers.
**Desire**	To understand the motivations behind pursuing digital transformation and the perceived benefits.
**Knowledge**	To gather information on existing knowledge and available guidelines to support digital transformation.
**Ability**	To explore the actions and measures taken to facilitate the adoption of digital technologies by healthcare professionals.
**Reinforcement**	To evaluate the metrics and KPIs used to measure the success of digital transformation initiatives.

Since the study focused on hospital managers’ perceptions of change management practices and did not involve patients, clinical data, or any sensitive personal information, formal ethics approval was not required under institutional guidelines. The study protocol was reviewed and confirmed as exempt from full ethical review by the Ethics Committee of University of Aveiro, in accordance with national and European data protection regulations (GDPR, Regulation (EU)). All participants received written information about the study’s objectives and procedures before the interviews. Informed consent was obtained from each participant prior to participation, and their consent was verbally confirmed and audio-recorded at the beginning of each interview. Participation was entirely voluntary, and confidentiality and anonymity were assured throughout the process. No identifiable personal data were collected, and all records were anonymised prior to analysis.

***3.2.2.2 Data analysis*:** The interview data were analysed, ensuring practical alignment with real hospital contexts.

To ensure transparency and methodological rigour, the analysis followed a structured coding process. The transcripts were coded using an initial deductive codebook informed by the theoretical roadmap, while additional codes were added inductively during the close reading of the interviews. The final themes and categories resulting from this coding process are summarised in Table 5.

**Table 5:** Themes and categories identified through thematic content analysis of the interview transcripts.

Theme	Category/ Subtheme	Description/ Interpretation
Humanisation in Digital Transformation	Patient-centred care	Emphasises that digital transformation should strengthen, not weaken, human connection in care delivery.
	Staff empowerment	Reflects the need to engage and support staff during technological transitions.
Organisational Change Management	Leadership and culture	Underlines leadership as a driver for successful change adoption.
	Communication and resistance	Highlights the role of transparent communication in reducing resistance.
Technology Acceptance	Perceived usefulness	Indicates that perceived value influences acceptance of new technologies.
	Ease of use	Demonstrates the importance of usability and tailored training.
Efficiency and Performance Outcomes	Process improvement	Shows tangible operational benefits linked to transformation.

In order to draw conclusions from the information obtained from the practical perspective, a thematic-categorical content analysis of the transcribed interviews was carried out. The aim of this analysis technique is to extract meaning from texts that deal with a particular subject under study. Thus, the usual stages of content analysis were followed, which consist of: organizing the material and defining the pre-analysis procedures; identifying emerging concepts through textual interpretation during the exploration phase; and finally processing and interpreting the results. Thematic saturation was reached after the sixth interview, as no new themes or categories emerged thereafter.

Saturation was assessed during coding by tracking the emergence of new codes and themes across consecutive interviews. After interview 6, no new themes were identified and subsequent interviews (7–8) served to confirm the stability of the thematic structure. Stability was evaluated based on (i) repetition of previously identified categories, (ii) absence of novel conceptual dimensions relevant to the roadmap components, and (iii) convergence in reported barriers/enablers across participants from different regions and regimes.

Table 6 summarises thematic stability by indicating when each ADKAR pillar first emerged and illustrating that later interviews reiterated the same pillars. In line with our analysis, no qualitatively new thematic directions were observed after the sixth interview, and the final interviews primarily reaffirmed previously identified perspectives.

**Table 6:** Evidence of thematic stability across interviews (ADKAR pillars).

ADKAR pillar	First observed (interview)	Illustrative early quote (translated)	Confirming later quote (translated)
Awareness (need for change)	M1	M1 “In Portugal, projects that tend to take a long time to be implemented face resistance. It is necessary to adapt planning to the Portuguese context, focusing on short timelines and quick results.”	M7 “When we only apply technology to modernise, without considering the main objective, instead of calling it a technological solution, people call it a technological burden.”
Desire (desire for change)	M2	M2 “If we applied the same effort and commitment we use to sell a new product to a customer to promoting changes in work inside the organisation, we would have different results.”	M8 “The reason for digitalisation is: whoever does not adapt to the digital world we live in does not survive.”
Knowledge (knowledge needed for change)	M2	M2 “Success depends on appropriate training, effective change management, and active involvement of employees.”	M7 “Digital transformation, instead of being seen as progressive leverage, is seen as a barrier to change. It is seen as a competing force against my role. That is why we increasingly see healthcare professionals resisting the internalisation of digitalisation—because, in reality, this is seen as a force that goes against helping us become better at what we deliver.”
Ability (capabilities of professionals)	M3	M3 “By disseminating results throughout the process, we will end up creating that culture change. Instead of being top-down (Ministry of Health) to bottom-up (health professionals), I think it will be more bottom-up. Because examples start to appear, and top leadership will have to realise that this change to digital is necessary.”	M6 “The more people understand and perceive the advantages of implementing these processes, the lower the resistance will be. If we ask people to leave their comfort zone—to change procedures/processes and work habits—people will join in without resistance if they understand that the change will bring advantages both for them and for the institution.”
Reinforcement (mechanisms to reinforce change)	M2	M2 “Cultural change should happen gradually and be slowly integrated into organisations. Digitalisation will help evolution, but organisational culture should not be changed abruptly.”	M7 “Most people are not willing to go on this journey because they were not even included in the initial phase of conceptualisation. Therefore, they are not connected at all to the technological solution. It is necessary to keep people involved from the beginning and accompany them throughout the whole process.”

***3.2.2.3 Sample characterization*:** In this subsection, we present only the characterisation of the interview sample. Analysing the profile of the eight interviewees, we can see a diversity of occupational, institutional and geographical profiles. Starting with the demographic data, the predominant age range of the interviewees is between 40 and 60, suggesting solid professional experience accumulated over the years. Concerning professions, we noted a variety of leadership positions in healthcare organisations, both public and private. The presence of interviewees from both public and private institutions suggests a comprehensive view of the practices and challenges faced by the healthcare sector in Portugal, providing a solid basis for comprehensive analyses and insights into the country’s healthcare system. In terms of geographical distribution, the interviewees are spread across different regions from the north to the south of Portugal, such as Porto, the Algarve, Coimbra, Lisbon and Braga, reflecting a representative sample of the country’s regional diversity. Additionally, the interviewees were chosen because of their existing experience in digital transformation, helping to provide valuable insights into the challenges and best practices in this process. The sample was selected for convenience. Table 7 shows the profile of each of the interviewees.

**Table 7:** Profile of the interviewed individuals in general management positions.

Designation	Region	Regime (Public/Private)	Profession	Age
M1	Porto	Public	Director of the Data Intelligence Service	40-50
M2	Porto	Private	Hospital Director	50-60
M3	Algarve	Public	Chairman of the Board of Directors	40-50
M4	Coimbra	Public	Chairman of the Board of Directors	40-50
M5	Coimbra	Public	Hospital Administrator	50-60
M6	Porto	Public	Executive Board Member	40-50
M7	Lisbon	Private	Director at Value Based Healthcare	40-50
M8	Braga	Public	Chairman of the Board of Directors	40-50

Although the study involved eight interviews, we observed thematic saturation by the sixth interview, with the final two interviews serving to confirm the stability of the identified themes. Participants were selected based on their managerial roles and direct involvement in digital transformation initiatives, ensuring information-rich cases relevant to the study objectives.

A small, information-rich sample was considered appropriate because the study was to refine a preliminary roadmap using expert managerial insights rather than to estimate prevalence or produce statistical generalisation. Participants were selected to maximise heterogeneity across regions and governance regimes (public/private), ensuring exposure to varied implementation realities”.

## 4. Results

This section is divided into two different subsections. The first subsection addresses **RQ1** by presenting an initial roadmap developed based on insights from the theoretical research, practical observation in real organisational settings, and informal conversations with domain experts involved in digital transformation projects. The second subsection addresses **RQ2** by presenting the final version of the roadmap, incorporating adjustments derived from the empirical component (semi-structured interviews with Portuguese hospital managers).

### 4.1 First roadmap for digital transformation with human focus based on theoretical and contextual research

As mentioned above, to establish a roadmap for digital transformation, it is crucial to recognize the pivotal role of human resource practices in planning and implementing digital transformation initiatives.

A fundamental component of digital transformation is establishing, enhancing and nurturing a digital culture “understood as a set of values and characteristic behaviours, at personal and organisational levels, driving new digitally enabled ways of thinking, working and interacting with the customer, among employees and business units, and with new digital tools” [86]. So, it is necessary to be careful about people and their feelings regarding this transition.

Taking into account the insights gleaned from the literature, it was possible to come up with a preliminary version of a roadmap for digital transformation focusing on the human component, which includes six phases (see Table 8).

**Table 8:** Roadmap phases.

	PHASES	EXPLANATION
I	Five lean principles for the hospital	The five lean principles are employed in the healthcare sector to enhance patient value by engaging in continuous improvement. This is accomplished by aligning with patient needs, cutting down on waste, and bolstering the efficiency and effectiveness of healthcare delivery [92].
II	Gap between what is real and what is desired	By defining the ideal, it is possible to understand the gap between what is real (where we are) and what is desired (where do we want to go?). At this stage, process improvement is considered a key factor in achieving the goal of adding value to the patient.
III	Which processes to digitalise	During this change, it is imperative to understand which of the processes to be improved and digitised will have the greatest impact, considering not only the organisation’s long-term vision, but also the resources available (human, investment, time, etc.). These will be the priority processes [93].
IV	AS-IS and TO-BE processes	Next, the modelling of theprocesses selected in the previous step should be carried out in two distinct phases. It begins with mapping the AS-IS processes, which consists of recording the processes in their current form. After this stage, the TO-BE processes are defined, outlining how they should be structured in the future [94]. a. When transitioning from the AS-IS model to TO-BE, it is crucial to initially adopt the lean methodology when creating the new processes, to eliminate inefficiencies and increase their operational efficiency, thus maximizing the value provided to the patient. Acero et al. [92]points out that lean principles support digital transformation in healthcare. Some authors even argue that lean can be seen as a prerequisite for introducing technology into processes, so as not to digitise waste [95–97]. b. Next, digitalisation should be implemented as a mechanism to increase the operational efficiency of processes. This reduces errors, improves execution speed and facilitates monitoring, resulting in a more agile service. c. During this digitalisation process, it is crucial to understand the need for interoperability between systems, since the degree of interoperability of IS is important for assessing the hospital’s degree of digital maturity [98]. d. Finally, it is essential to select key performance indicators (KPIs), the operational health outcomes to monitor processes, ensuring that they reflect clear and measurable objectives, aligned with organisational priorities/goals. Understanding and effectively communicating these KPIs is fundamental for the team, and regular analysis of the data collected is essential to maintaining the quality and efficiency of the health service.
V	Action plan	Once the foundations have been laid, it is essential to draw up a detailed action plan. This plan should articulate the steps and timelines, assign responsibilities and allocate the necessary resources. It must be ensured that the plan is flexible enough to adapt to unforeseen changes and challenges, while remaining focused on the long-term vision and the mission of adding value to the patient.
VI	Change management methodology	During hospital digitalisation, it is essential to adopt change management focused on both processes and people to ensure a successful transition. Schulte et al. [99] highlight the importance of the well-being of healthcare professionals in this complex and constantly evolving scenario. Thus, employees who remain receptive during transformation initiatives in the organisation are better prepared to absorb and adopt new work concepts and methodologies [35]. As mentioned in section 2.3, the change management methodology to be used is ADKAR.

**Fig 4:**
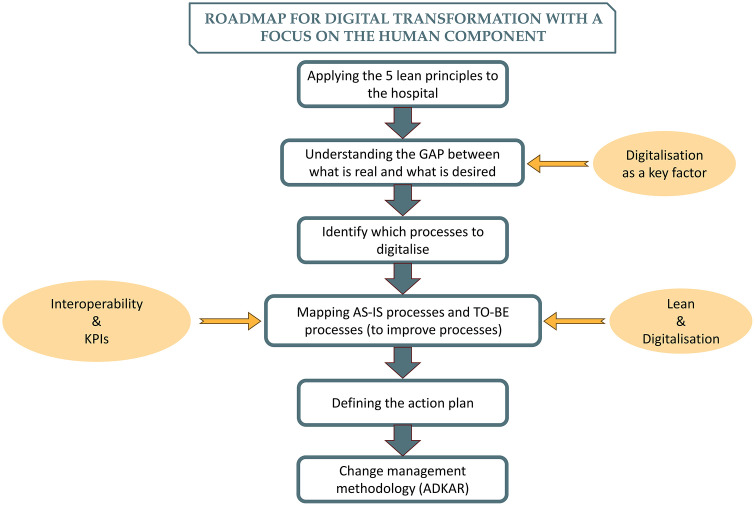
Initial roadmap for planning and implementing digital transformation with a human focus.

Fig 4 shows the proposed change management tool, based on the theoretical research, which was the basis for the empirical study.

### 4.2 Final roadmap for digital transformation with human focus based on empirical component

This section presents a revised version of the roadmap introduced in Section 4.1, now strengthened by the insights obtained from interviews with hospital management professionals.

#### 4.2.1 Experts’ perception of a possible roadmap for digital transformation in healthcare.

The interviews with hospital managers provided a practical understanding of the digital transformation initiatives implemented in Portuguese hospitals, helping to refine and adapt the roadmap initially developed.

In general, the professionals agreed with the approach taken to the roadmap, highlighting and valuing the stages presented, the techniques used in each stage and the consideration of the human component. In addition, several professionals reported positive previous experiences with the lean methodology, highlighting how its application had contributed to the mapping and optimization of processes. They also emphasized that the digitalisation process was tackled efficiently within the hospital, indicating that applying these practices was perceived as beneficial for the institution. The interviewees’ feedback reinforces crucial elements for the success of digital transformation in the hospital environment: the importance of managing organisational culture and continuous communication. Below are some of the topics highlighted by the managers regarding the roadmap, and the improvements/opinions they suggested.

- **Inclusion of health professionals (M1, M2, M4, M5, M6, M7):** The importance of including healthcare professionals from the start of the project and throughout is highlighted as an important component of the roadmap. This is intended to foster stakeholder acceptance and support, improve understanding of the objectives, and strengthen their sense of involvement in the project. This initial involvement is crucial to the roadmap’s success. [“In the human component there are two crucial aspects - the involvement of the people who are going to benefit from TD is essential and we must allow these people to be on the ground and build the transformed process. They must feel that the work is well done and that they have contributed to the process; another important issue is the involvement of top management” (M4).
- **Promote the action plan (M2, M5, M6):** It is important to “sell” the action plan as part of the change to guarantee results. [“You must have the ability to influence, to make people see what they haven’t seen yet” (M5)].
- **Organisational culture management (M3, M4, M5, M7):** It is suggested that the change in organisational culture occurs throughout the process, with the dissemination of positive results to facilitate the acceptance of new technologies. Bottom-up implementation can be more effective by sharing successes and improvements achieved, to trigger change in the top healthcare management in Portugal. [“It is crucial that the Ministry of Health recognizes and supports the importance of digitalisation to facilitate a broader transformation” (M3). “Organisational culture is difficult to change quickly and cannot be imposed externally. Small changes can be introduced gradually but will take years to be recognized. The advent of technologies can help, but cultural change is more about the essence of the organisation. The transformation of culture must be gradual and not just focused on digitalisation, to avoid bigger problems” (M2). “An organisational culture medication component is needed” (M7)].
- **Roadmap format (M3, M4, M6):** The format of the roadmap should evolve from linear to circular, allowing for a more effective representation of the idea of continuous improvement. This circular approach highlights the importance of constant adjustments, ensuring that the digital transformation process continually adapts to the needs and feedback of those involved, promoting a culture of innovation and efficiency within the institution. [“I wouldn’t change anything in terms of content, but I would give it a more circular note, as a result of the dynamism that the plan has to have” (M4). “Continuous feedback from professionals should be incorporated in order to adjust and improve the stages of the roadmap. In addition, allow the process to be circular, so that it can be revisited somewhat frequently, to enable changes, adjustments and corrections to be made to certain steps” (M6)].

It was proposed that the roadmap be restructured into a production chain flow format, which includes input, processing and output stages. “At first it will be ADKAR itself and the assessment of the organisational culture as an initial diagnostic factor to assess where that organisation, those people are at, before entering the processing mechanism, in order to have a direct output of what can be my KPIs. In other words, understanding what they need, understanding how they are, understanding how we are going to involve them?” (M7). At the processing point, people’s culture and what they need is modelled, and the entire process is carried out. KPIs should be measured both in the intermediate phase and at the end to monitor and evaluate the progress of the digital transformation.

- **Value in healthcare (M7)**: It is necessary to define what is value in health, value-based healthcare in the light of Michael Porter’s writings. In his book [100], he proposes a transformation in the healthcare system, arguing that competition should be based on creating value for patients, defined as the relationship between health outcomes and costs. They argue that the quality of health outcomes should be the focus, promoting a shift from volume-based to value-based competition. This involves a paradigm shift aligning all agents in the healthcare system, including providers, payers and patients, to prioritize efficiency and effective results.
- **Internal communication (M6):** Effective internal communication is fundamental to the success of the digital transformation, and there must be communication between all levels of the organisation, ensuring that everyone involved is informed, committed and aligned with the project’s objectives. All of this promotes collaboration and strengthens cohesion between teams. [“It’s essential to include an internal communication component to ensure that everyone involved clearly understands the advantages of joining the digital transformation. The more effective this communication is, and the better-informed people are about the benefits of the action, the less resistance they will have” (M6).]
- **KPIs and health outcomes (M5, M7):** It is important to note that KPIs and the measurement of health outcomes are essential for the continuous management and updating of the digital transformation project over time. Knowing the state of the process is crucial; ultimately, it is always necessary to measure everything possible. Table 9 shows the KPIs mentioned by the managers interviewed.

**Table 9:** KPIs for evaluating the success of digital transformation mentioned by interviewees.

KPI	Interviewee
• Amount of paper used; • Number of processes that are already supported by technology; • Number of interactions that exist with the systems; • Number of difficulties that arose in the process.	M2
• Timetables for correcting errors and integrating functionalities; • Number of consultations and exams carried out at the kiosks versus in person; • Analysis of the interoperability maturity level.	M3
• Measure of patient satisfaction with the services provided; • Operational efficiency and quality: ◦ Production indicators: number of services delivered. ◦ Financial indicators: profitability, costs and expenses. ◦ Performance indicators: operational efficiency, response times, treatment success rate. • Patient-team relationship: ◦ Level of closeness between the patient and the team: assessment of patients’ trust and safety in relation to the medical team. ◦ Easy access for patients to a multidisciplinary team: measure of how easy it is for patients to access different specialists. • Systems management and guidance: ◦ Possibility of the patient reporting unpleasant symptoms: frequency and effectiveness in responding to reported symptoms. ◦ Rapid guidance: average response time for guidance after reporting symptoms. ◦ Rapid clarification of doubts: average time taken to clarify patient doubts. • Rate of satisfaction and gratification of health professionals with innovative projects. • Resource management and adverse events: ◦ Efficiency in resource management: indicators of efficient use of available resources. ◦ Possibility of anticipating and better controlling adverse events: frequency and effectiveness in anticipating and controlling adverse events. ◦ Emergency resource minimization: rate of reduction in the use of emergency services.	M4
• Adherence rate to the new solutions implemented (platform); (M1, M5, M6, M7) • Rate of use of platform to make clinical decisions; (M7) • Usefulness of information for decision-making (the information generated by this platform was useful for my decision-making); (M7) • Rate of reduction in administrative bureaucracy with the use of this platform. (M7) • Connecting professionals to the usefulness of a platform (M7)	M1, M5, M6, M7

With these suggestions, the roadmap was revised to incorporate the suggestions of the managers interviewed, considering their needs and good practices. Section 4.2.2 will explain the final roadmap.

#### 4.2.2 Final roadmap.

Taking the interviewees’ inputs into consideration, the model was updated to a circular version (with the same steps described above in Table 8), incorporating concepts of managing and measuring organisational culture, as well as change management with continuous evolution, reflecting the main focuses of the professionals involved. Measuring organisational culture is crucial for selecting appropriate actions to support staff and teams in achieving the intended goals. In addition, the model was given the structure of a production flow that includes input, processing and output stages. Special focus was given to KPIs as metrics for evaluating the success of the digital transformation. In addition, the concept of value in healthcare was defined as explained by Michael Porter.

To this end, as mentioned by several interviewees, it will be important in this transformation process to build one or more teams focused on digital transformation, empowered and multidisciplinary teams that have the autonomy to make decisions and implement solutions.

**Fig 5:**
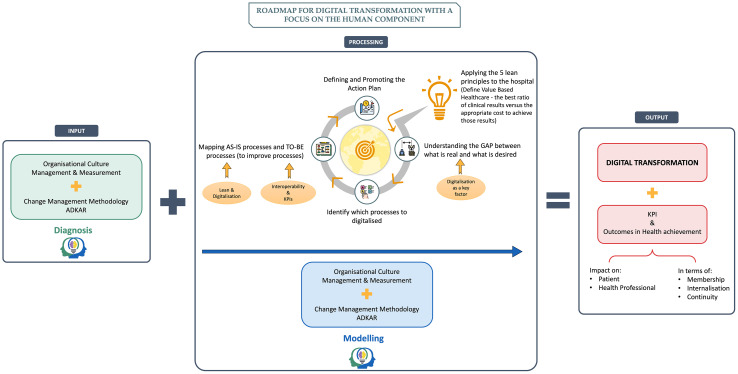
Final version of the planning and implementation roadmap for digital transformation with a focus on the human component.

Fig 5 shows the final version of the roadmap for the hospital’s digital transformation, with a focus on the human component, a change management tool.

To strengthen the operationalisation of humanisation within the roadmap, we make explicit how key roadmap elements can support human-centred outcomes through plausible mechanisms. Table 10 maps selected roadmap elements to the intended humanisation dimensions (patient and staff experience) and to example indicators, clarifying that these represent measurable targets for monitoring and future evaluation rather than empirically confirmed effects.

**Table 10:** Mapping of roadmap elements, mechanisms, humanisation dimensions, and example indicators.

Roadmap element	Mechanism (how it can support humanisation)	Humanisation dimension (observable)	Example indicator(s)
Workflow redesign (lean, standardisation)	Reduces administrative friction and non-value tasks; and protects time for care interaction	• Staff workload and wellbeing • Patient communication and relational quality	• Time spent on administrative tasks • Perceived workload/admin burden • Patient communication/experience item or index
Interoperability and continuity of information	Reduces repeated data entry and interruptions; and, improves coordination and relational continuity	• Relational continuity • Trust • Reduced staff stress	• Interoperability maturity level • Repeated documentation proxy • Patient trust/relational quality item or index
Participation/co-design and engagement practices	Increases ownership, voice, and clarity; and, supports psychological safety	• Participation • Psychological safety • Meaningful work	• Participation rate in co-design/change sessions • Psychological safety score • Engagement/meaningful work score
Training and ongoing support	Improves confidence and reduces anxiety related to digital tools	• Staff wellbeing • Perceived competence	• Training completion rate • Perceived usefulness of training • Self-efficacy/adoption confidence item
Governance, communication and role clarity	Reduces ambiguity and change-related stress; designed to improve trust and coordination	• Psychological safety • Trust • Coordination quality	• Perceived quality of communication about change • Role clarity item • Reported change-related stress item or index
Digital inclusion measures	Prevents exclusion and uneven burden; protects dignity and autonomy	• Equity/digital inclusion • Dignity/autonomy	• Digital inclusion proxy (need for assistance/access barriers) • Patient autonomy item

Table 11 gives a brief explanation of the different phases of the final version of the roadmap, with some specific KPIs to assess progress and performance of the implementation in each phase, covering both operational outcomes and human-centred dimensions (patient experience and staff experience).

**Table 11:** A brief explanation of the final version of the roadmap and some KPIs associated with each task.

Step	Task	Explanation	KPI
Input	Organisational culture management & measurement (diagnosis)	Evaluate and measure the current organisational culture to establish a baseline and identify areas for change, including staff experience and readiness for human-centred digital transformation.	• Results of questionnaires assessing organisational culture • Professional satisfaction index • Percentage of professionals who remain in the hospital over time • Measuring the presence of leadership. • Staff wellbeing (short validated wellbeing measure, examples include WHO-5, or internal index; to be selected for the local context) • Sick leave/ turnover related to workload/stress **•** Psychological safety score (short validated psychological safety measure, examples include Edmondson or internal index; to be selected for the local context)
	Change management (diagnosis)	Evaluate current management practices, including readiness, perceived barriers, and staff concerns that may affect human-centred adoption.	• Results of questionnaires assessing the willingness and ability of professionals to change; • Number of management practices which will benefit from change • Number of management practices identified as ineffective • Number of interactions with systems • Number of processes already supported by technology • Amount of paper used (indicates successful adoption of digital systems) • Readiness-to-change score (staff survey/index) • Perceived quality of communication about the change (staff survey score) • Participation rate in change-related meetings/training/co-design sessions (%)
Processing	Applying the 5 lean principles	Using lean principles to understand and define value in health, reducing non-value activities to support time for care and human interaction.	• Measurement of how process improvements influence health outcomes • Evaluation of patient satisfaction with the care received, weighted by health outcomes and associated costs • Example: number of consultations and exams carried out at the kiosks versus in person • Time spent on administrative tasks per professional (before/after) • Time spent in direct patient interaction (self-report or time-motion proxy)
	Understanding the GAP	Analyse the differences between the current state and the desired results to prioritize changes, including gaps affecting patient experience and staff wellbeing. Understanding how digitalisation can be a key factor in achieving the goal.	• Number of critical gaps identified between the current and desired state • Evaluation of the impact of digitalisation on the reduction of gaps • Interoperability maturity level analysis. • Priority score incorporating “human impact” alongside feasibility (e.g., impact on patient interaction and staff workload)
	Identify which processes to digitalise	Select the main processes that would benefit from digitalisation, and that bring the most benefits to the institution/people, explicitly considering human impact on patients and staff.	• Number of processes already supported by technology • Measurement of the expected impact of digitalisation on these processes (ICE – impact, cost, easiness) • % of prioritised processes assessed for human impact (patient interaction, staff workload)
	Mapping AS-IS processes and TO-BE processes	Documenting current processes and outlining future processes, based on lean to eliminate/mitigate waste and digitalisation to automate tasks, while safeguarding patient–professional interaction and reducing staff burden.	• Percentage of processes mapped, of those identified to be digitised • Number of improvement opportunities identified from AS-IS for the TO-BE process • Timetables for correcting errors and integrating functionalities **•** Number of workflow changes explicitly designed to reduce staff cognitive load (count/track)
	Defining and promoting the action plan	Create a detailed action plan based on the needs identified, including communication, training and engagement actions to support human-centred change.	• Percentage of planned tasks that have been described in detail and scheduled • Level of stakeholder support and commitment to the defined plan • Teams’ understanding of the benefits of digitalisation • Connecting professionals to the usefulness of a platform • Training completion rate (by role) and perceived usefulness of training (feedback score) • Engagement/participation rate in change activities (co-design sessions, workshops) (%)
	Organisational culture management & measurement (modelling)	Manage and measure the organisational culture, to align the company’s culture with its goals in relation to digital change, monitoring staff experience and cultural alignment over time.	• Measure whether the organisational culture is aligned with the digitalisation/change goals • Rate of satisfaction and gratification of health professionals with innovative projects • Trend in staff wellbeing and psychological safety over time
	Change management (modelling)	It involves planning and implementing organisational changes by creating strategies that ensure that the changes are effectively adopted by professionals, minimizing resistance and maximizing the positive impact on health value, including sustained engagement and reduced change-related stress.	• Percentage of measures implemented by managers to ensure the success of the change • Measurement of the reduction in resistance to change by professionals after implementing the strategies • Monitoring the acceptance of digital technologies over time to assess changes in user attitudes • Take-up rate of the new solutions implemented • Sustained adoption rate over time (e.g., 3–6 months after rollout) • Perceived change-related stress (single item or short scale; staff survey score)
Output	KPI measurement and achievement of digital transformation	Measurement of KPIs that assess the impact on two main groups: the patient and the healthcare professional: • Membership: assesses people’s acceptance of and involvement with new digital technologies • Internalization: measures the extent to which digital practices are internalized by healthcare teams and patients • Continuity: try to ensure that engagement with digital solutions is sustainable over time, and whether digitalisation remains aligned with humanisation goals (patient experience and staff experience). Human-centred outcomes are monitored through patient experience (communication, dignity, trust, continuity) and staff experience (wellbeing, workload, psychological safety, meaningful work and participation).	• Percentage of acceptance and use of new technologies by patients and professionals • Measuring the impact of new technologies on patients’ clinical outcomes • Percentage of departments that have adopted new digital technologies • Measuring the digital skills acquired by healthcare professionals and patients after implementing new technologies • Evaluation of patient autonomy using digital health management solutions • Measuring the frequency of use of digital solutions by patients and healthcare professionals • Evaluation of user satisfaction with the technologies implemented, by means of regular questionnaires • Measuring how digital technologies have impacted clinical efficiency in terms of time saved on administrative tasks and care • Measuring the improvement in communication between different departments due to the implementation of digital solutions (interoperability) • Perceived workload/administrative burden (staff survey score) • Patient experience: communication/respect/dignity item/index • Relational quality and trust (patient-team relationship score) • Staff engagement and meaningful work (engagement score or job satisfaction item)

Overall, despite some variations in focus, participants shared a common understanding that digital transformation in hospitals must be carefully planned and implemented to balance technological efficiency and humanisation. A general consensus emerged around the importance of leadership, communication, and staff engagement as key enablers of successful change.

## 5. Discussion and reflections on the results

The digital transformation takes place at various levels and therefore requires an integrated view of the organisation and its decision-making processes. It is imperative to ensure that planning and implementation consider all organisational levels, since this type of transformation can reshape roles, routines and coordination mechanisms. In addition, it is necessary to understand that digital transformation can lead to changes in organisational culture and in the way, work is carried out. Therefore, to enter a digital transformation process, it is first necessary to understand the impact of digital transformation on the healthcare ecosystem and what planning, implementation and management mechanisms need to be in place to correctly introduce digital transformation practices.

This study presents an exploratory, context-sensitive roadmap to support the planning and implementation of hospital digital transformation, grounded in literature and contextual inputs and empirically refined through interviews with Portuguese hospital managers (with a focus on humanisation, organisational culture and change management). As the roadmap has not yet been piloted in real-world settings or systematically compared with existing implementation frameworks, effectiveness and comparative value should be assessed through future piloting and evaluation with broader stakeholder involvement.

Based on the analysis of the interview responses, several reasons can be identified that motivated the digital transformation process in the hospital. A central reason highlighted by several managers (M1, M2, M5, M6) is the increase in process efficiency, economic efficiency and the need to continuously improve the quality of health services, as mentioned by Dal Mas et al. [11]. Digital transformation is also seen as a way of improving the provision of a quality service, to be aligned with the growing demands of users and society, providing a better service to users (M5). This transformation aims to facilitate the work process (M2), bring the group’s clients closer together by centralizing their information (M2), and broaden the channels of communication with clients, both commercially and clinically (M2).

Another important point mentioned as a critical reason for digital transformation was the need to develop interoperability with other institutions, in order to improve the quality and quantity of clinical information exported, which was highlighted as essential for offering better patient support and, consequently, improving the quality of healthcare (M4); the literature mentions interoperability as an essential pillar for improving the quality of care. [98]. In addition, another topic mentioned was the need to adapt to the digital environment as a way of staying “alive” and “surviving” in the hospital market (M8).

It is interesting to note from the analysis of the interviews that there is a duality in the digital transformation: while it drives innovation and efficiency, it also presents significant challenges for the workforce, especially about retraining and adapting to the new roles that emerge. Thus, the transition not only requires investment in technology, but also in training and retraining programmes to mitigate adverse social impacts and ensure that technological progress benefits society. This analysis, which came from the motivations for hospitals to start digital transformation, was the “increased flow of demand from users” (M7), and “clearly the lack of human resources” (M3). Another interviewee highlights a recurring concern among health professionals: “one of the things that scares professionals at the moment is the certainty that some professions and some medical specialties are going to disappear with these digitalisation processes in the sense of replacing or integrating artificial intelligence into these contexts, but it’s clear that there are others that are going to emerge and that are going to, let’s say, gain different relevance and another level of importance”. What’s more, he stresses the importance of considering that “there are people who can be retrained and others who will not be retrained for new positions. This will be a huge challenge for the social protection system. (M5)

In line with this, another idea put forward by the professionals is that training is the basis for digital transformation, as it will also be in the case of a paradigm shift, which in many cases can be from 2.0 or 3.0 to 5.0. However, training is not solely or primarily about formal education. It should focus on fostering an institutional culture that embraces innovation. This verifies what is mentioned in the literature on training, in that it makes people feel less threatened and more prepared for change [72,74].

One of the professionals mentioned that there are differences in the results when there is a clear objective for the digital transformation, or when the intention is just to modernize without a definition of the purpose, in his statement: “When we just apply technology to modernize, not taking into account the main objective, instead of calling it a technological solution, people call it a technological burden” (M7)*.* This corroborates one of the aspects that led to this study – the fact that it is necessary to correctly identify the problems to be solved to develop robust technologies whose implementation effectively contributes to improving health outcomes and the efficiency of health services. This shows once again the importance of structured change based on the roadmap.

Another idea mentioned by the interviewees during the digital transformation process is the need to keep people involved at all stages of the process to foster commitment and active participation. As one of the managers said, “The main change is people/involvement, everyone, everyone, everyone, quoting Pope Francis at WYD 2023” (M4). “In the human component there are two crucial aspects – the involvement of the people who will benefit from the digital transformation is essential and we must allow these people to be on the ground and build the transformed process. They have to feel that the work is well done and that they have contributed to the process; another important issue is the involvement of top management” (M4). This verifies the ideas presented in the literature that it is crucial to involve people from the start of the transformation to awaken people’s commitment to change [35,71–74]

It was noticeable that one of the topics mentioned as necessary factors for achieving a successful digital transformation in hospitals is keeping people inspired. As one of the interviewees mentioned:

The biggest challenge is undoubtedly convincing people, teams, that it really is worth doing things differently and going through the pain of changing processes. You have to have the ability to influence, to make people see what they haven’t seen yet … Leadership needs to have a great ability to influence. They need to create memories of the future in people about what they are going to achieve with the digital transformation, in other words, get them to go ahead, see how they could be working ahead, imprint that memory on them and then make them go through the process to get there. (M5)

In addition, it was also mentioned that: “If we put exactly the same effort and commitment that we put into selling a new product to the customer into promoting changes at work within the company, we’ll get different results” (M2). This message corroborates what has been found in the literature about the extreme importance of the role of supportive leadership in this transformation [27,35,73,74].

Another of the managers interviewed highlighted the critical importance of ensuring that healthcare continues to value the patient experience, respecting their dignity and providing a healthy working environment for professionals. As highlighted by Gaspar et al. [30], professional satisfaction influences organisational results and, therefore, the focus on the human component and humanized care becomes essential. So, as we move towards an increasingly digital future, let us not lose sight of the importance of proximity and empathy in healthcare. The integration of new technologies must be accompanied by a continuous effort to preserve effective communication, multidisciplinary teamwork and a care network that puts the person at the centre.

To paraphrase Sir William Osler (1849–1919), a seminal figure in modern medicine: “Just as important as knowing the disease that man has, is knowing the man who has the disease”. “Even with technological advances, this humanism can never be lost” (M4). What is more,

In the future, the biggest challenge will be not to lose focus on the person. Let’s not forget, however, that modernity, in which AI is embedded, cannot make us forget our commitment to humanisation. Responses that value the patient’s experience, the proximity of care, full respect for the person and their dignity and, on the professionals’ side, healthy, safe and sustainable working environments are essential, with communication, multidisciplinary and networking being fundamental premises to preserve. (M4)

The modernization of medicine must therefore balance technological innovation with maintaining the humanistic values that form the basis of a truly effective health system, and this issue has also been discussed in the literature by Wanasinghe et al. [13].

## 6. Final remarks

### 6.1 Conclusion

For hospital digital transformation to be successful, it is essential to follow a clear organisational direction, and set priorities, and adopt an integrated approach that considers technology, processes, people and organisational culture.

The centrality of human beings in digital transformation is not only an ethical issue, but also an organisational and operational imperative one, and the need to develop a roadmap for digital transformation with a focus on the human component was recognized and was considered an essential aspect by the interviewees. The roadmap was adjusted to the needs of the hospital managers interviewed, as illustrated in Fig 5. This approach is in line with what Gilli et al. [27] state in their study, that people, rather than technology, drive digital transformation. The importance of developing the roadmap presented above is therefore clear.

**Fig 6:**
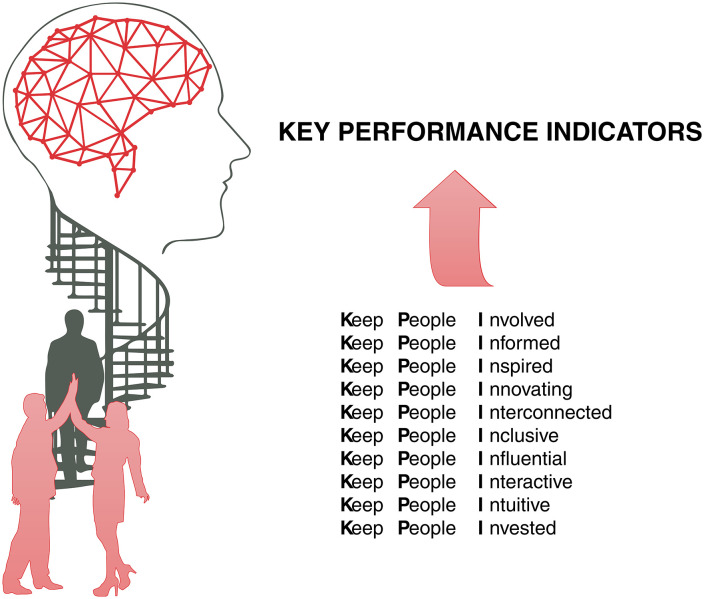
The human factor as a crucial element in the success of KPIs. Adapted from: Robertson Hunter Stewart.

As mentioned earlier, people are central to the success of the digital transformation, and this success is measured through KPIs. One of the key conclusions drawn from the interviews was: To guarantee the effectiveness of the KPIs and obtain satisfactory values for them, it is essential to keep people: involved, informed, inspired, innovating, interconnected, inclusive, influential, interactive, intuitive, and invested. In this digital transition, human capital has come to be seen as an enabler and catalyst for innovation. Fig 6 shows a playful diagram of this conclusion.

- **Involved**: “Keep People Involved” – Keeping people involved in all phases of the digital transformation process to foster commitment and active participation.
- **Informed**: “Keep People Informed” – Ensuring that everyone is up to date with the latest digital trends and practices for an informed transition.
- **Inspired**: “Keep People Inspired” – Constantly encouraging the team with visions of the future and innovations that show the positive impact of digitalisation.
- **Innovating**: “Keep People Innovating” – Stimulating creativity and experimentation to find new and improved digital solutions.
- **Interconnected**: “Keep People Interconnected” – Ensuring that communication and collaboration networks are optimized in the digital age.
- **Inclusive**: “Keep People Inclusive” – Promoting a culture of diversity and inclusion in the digital environment.
- **Influential**: “Keep People Influential” – Empowering employees to be agents of change and positively influence the direction of digitalisation.
- **Interactive**: “Keep People Interactive” – Providing digital interfaces and experiences that promote continuous interaction and involvement.
- **Intuitive**: “Keep People Intuitive” – Developing systems that are easy to understand and use, keeping the user experience at the heart of digital innovation.
- **Invested**: “Keep People Invested” – Cultivating a sense of ownership and commitment to the organisation’s digital goals.

Furthermore, this study clarifies that the balance between humanisation and digitalisation is not a conceptual abstraction, but a practical principle incorporated throughout the roadmap. Each phase of the proposed model operationalises this balance, linking technological initiatives to cultural diagnostics, team involvement, communication, training, and continuous measurement of results. By integrating human-centred indicators alongside operational KPIs, the roadmap provides a structured basis to assess whether digital transformation remains aligned with humanisation goals in hospital settings. This addresses the study’s initial intent by offering a feasible way to support and monitor this balance in real hospital contexts.

Accordingly, the roadmap should be interpreted as a proposal designed to support humanisation through measurable indicators, while further piloting is needed to assess real-world effects.

### 6.2 Contributions and implications

- **Theoretical implications:**

This paper contributes to the literature by presenting an implementation-oriented roadmap for digital transformation, grounded in theory and empirically refined, with a focus on the human dimension. Proposed as a change-management support tool, it is intended to inform implementation planning rather than to report a tested intervention, as it has not yet been piloted or validated in real-world settings. By addressing a recognised gap, the study advances research on change management and digital transformation in healthcare and highlights priorities for future empirical validation.

- **Practical implications:**

By incorporating human component perspectives into an implementation roadmap, healthcare institutions may better navigate the organisational complexities of digital transformation while maintaining a focus on humanisation. The roadmap supports the structuring and sequencing of initiatives, stakeholder engagement, and monitoring through KPIs. However, implementation outcomes, adoption at the practice level, and effects on patient and staff experience require local tailoring and should be assessed through piloting and evaluation in real-world settings.

### 6.3 Limitations and future work

- **Limitations:**

The study relies on a small qualitative sample of Portuguese hospital managers (n = 8), so findings primarily reflect managerial perspectives within a specific context and may not represent frontline staff, patients, or other stakeholders; transferability requires local tailoring. Interview-based analysis may be affected by participant/researcher bias and by evolving policy and technology conditions. The roadmap is a theoretically informed and empirically refined planning proposal, not a tested intervention: it has not been piloted or compared with existing frameworks, so future evaluation is needed to assess effectiveness and comparative value.

- **Future work:**

Future work would include interviews with other stakeholders, such as doctors, nurses and patients, to get a more holistic view of digital transformation in the healthcare sector. In addition, we plan to implement/pilot the roadmap in a real-world context, conducting longitudinal studies to assess the roadmap’s long-term impact on healthcare institutions and adjusting approaches as necessary. Future work also plans to digitise the roadmap, as well as develop and document metrics and performance indicators to assess the effectiveness of digital transformation initiatives and the impact on the quality of healthcare services and patient satisfaction. In addition, the intention is to define a set of KPIs that should be measured as an “output” of the roadmap.
